# siRNA Treatment: “A Sword-in-the-Stone” for Acute Brain Injuries

**DOI:** 10.3390/genes4030435

**Published:** 2013-09-05

**Authors:** Andrew M. Fukuda, Jerome Badaut

**Affiliations:** 1 Department of Physiology, Loma Linda University School of Medicine, Loma Linda, CA 92354, USA; E-Mail: afukuda@llu.edu; 2 Department of Pediatrics, Loma Linda University School of Medicine, Loma Linda, CA 92354, USA

**Keywords:** brain, siRNA, RNAi, traumatic brain injury, stroke, ischemia, subarachnoid hemorrhage

## Abstract

Ever since the discovery of small interfering ribonucleic acid (siRNA) a little over a decade ago, it has been highly sought after for its potential as a therapeutic agent for many diseases. In this review, we discuss the promising possibility of siRNA to be used as a drug to treat acute brain injuries such as stroke and traumatic brain injury. First, we will give a brief and basic overview of the principle of RNA interference as an effective mechanism to decrease specific protein expression. Then, we will review recent *in vivo* studies describing siRNA research experiments/treatment options for acute brain diseases. Lastly, we will discuss the future of siRNA as a clinical therapeutic strategy against brain diseases and injuries, while addressing the current obstacles to effective brain delivery.

## 1. What is siRNA?

### 1.1. Discovery and Endogenous Function

The discovery of the ribnonucleic acid (RNA) interference mechanism through the activity of small interfering RNA (siRNA), also known as short interfering RNA, has proven to be a powerful molecular tool to study functions and properties of individual proteins. The potential usage of siRNA is no longer limited to dissections of molecular protein pathways, and the last decade has revealed new potential. siRNA shows promise as a powerful clinical tool targeting individual proteins that are known to cause certain pathophysiological conditions after disease and injuries [1,2,3].

**Figure 1:**
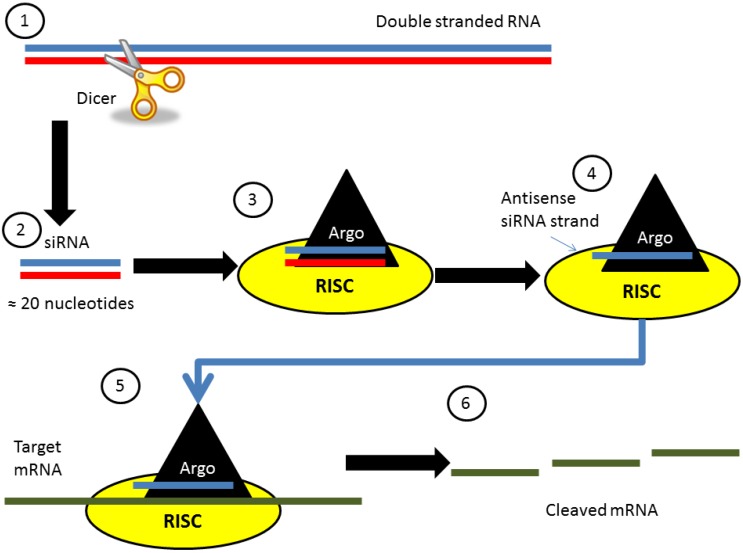
Molecular pathway for siRNA processing.

RNA interference is an endogenous mechanism present across phylogenetic groups, and has been described in humans, plants and animals. Generally, RNAs are best known for their role in gene expression and protein synthesis from DNA, such as messenger RNA (mRNA) and transfer RNA (tRNA). Although a single-stranded molecule in many of its biological functions, RNA can form intra-strand double helixes upon itself, which may trigger post-transcriptional gene silencing, also known as RNA interference. During interference, microRNA (miRNA) and/or siRNA molecules can inhibit gene expression, typically by causing the destruction of specific mRNAs. Dr. Fire and Dr. Mello received the Nobel Prize for the ground-breaking discovery of this RNA interference mechanism described in *C. elegans* [4], with later studies devoted to the discovery of specific molecular mechanisms of RNA interference processes [5]. Thus, only a very brief explanation of the siRNA pathway will be provided here (Figure 1), since our review will be focused on the possible use of siRNA in acute brain injuries.

siRNA is a short double-stranded molecule composed of about 20 nucleotide base pairs organized in corresponding sense and antisense strands. These short double-stranded molecules are produced as a result of an enzyme called Dicer [6,7], which cleaves a longer double-stranded RNA present in the cell cytoplasm. These double-stranded RNA composed of both the sense and antisense strand is a necessary trigger for the generation of siRNA. This was elegantly demonstrated by Mello and Fire, when phenotypic changes were observed in *C. elegans* following insertion of both the sense and antisense mRNA of a particular protein, but no changes occurred upon delivery of a single strand of either the sense or antisense strand [4,8]. Once siRNA is made, it enters a protein complex called the RNA induced silencing complex (RISC) where Argonaut 2 cleaves the sense strand away from the antisense strand. The antisense strand remaining within RISC is now free to target complementary endogenous mRNA for subsequent cleavage by the RISC-Argonaut2-siRNA complex, thereby interfering with the translation process of that specific protein [7]. This ultimately leads to significant down-regulation of the protein normally encoded by the targeted mRNA (Figure 1). This elegant yet simple mechanism allowed the advent of numerous basic science discoveries and research advances due to the specificity and potency of the RNA interference mechanism in targeting proteins of interest. Ongoing research interests include translational approaches of this newfound mechanism in clinical practice, and using siRNA as a therapeutic agent against diseases including acute brain injuries.

### 1.2. Clinical Treatment Tool

Because of its scientific promise and allure, synthetic siRNAs developed against diseases such as HIV, cancer, diabetes, and infection are currently in various phases of clinical trials, as reviewed by Burnett *et al*. [1,2]. For example, *vascular endothelial growth factor (VEGF*) or its receptor have been popular targets for siRNA to counteract overt angiogenesis present in diabetic macular edema, age-related macular degeneration, and cancerous solid tumors [2]. siRNA against *VEGF* for diabetic macular edema has completed phase II, and the clinical trial against solid tumors is now in a long-term phase I trial showing promising results [2,9].

However, to date, there has been no siRNA therapeutics in clinical trials for disorders or injuries of the central nervous system. In part, the lack of clinical translation regarding neurological disorders is due to the difficulty of effective siRNA delivery across the blood-brain barrier (BBB) and into desired brain regions. Additionally, there is a relative paucity of *in vivo* data using siRNA treatment following acute brain injuries, such as traumatic brain injury (TBI) and stroke. However, promising new developments and recent preclinical results may soon translate from the bench to the bedside, as discussed in the remainder of the review.

## 2. siRNA and Acute Brain Injuries

Although siRNA awaits application for treatment of acute brain injuries in humans, in recent years, several animal studies have been performed in which siRNA was used as a potential therapeutic tool in acute brain injury models. This section will focus on *in vivo* work in which siRNA was used as a therapeutic tool in models of acute brain injuries such as brain hemorrhage, brain ischemia, and traumatic brain injury (TBI).

### 2.1. Non-Traumatic Brain Hemorrhage Models

#### 2.1.1. Background

Intracranial hemorrhage or brain hemorrhage refers to bleeding that occurs either in the parenchyma of the brain (subarachnoid hemorrhage (SAH)), or the surrounding intracranial spaces (intracranial hemorrhage (ICH)), so named depending on its anatomical origin. Both ICH and SAH can arise from either a traumatic or non-traumatic etiology. Traumatic etiologies will be covered later in the review of traumatic brain injury (TBI). Non-traumatic ICH and SAH result from ruptures of intracerebral blood vessel due to a variety of other symptoms, such as hypertension and accumulation of beta-amyloid (amyloid-β or Aβ) angiopathy. Phenotypic changes in vascular properties may cause vessels to rupture and result in small bleeds or micro-hemorrhages. In the case of ICH, micro-hemorrhages cause hematomas, which contain inflammatory factors in direct contact with the brain parenchyma, leading to many dysfunctions and often resulting in permanent damage or neuronal death. In a similar harmful cascade, SAH promotes intracranial aneurysms leading to blood extravasation and damage in the subarachnoid space. ICH and SAH initiate many pathological molecular cascades such as apoptosis, BBB disruption, and edema, which can ultimately lead to chronic damage, disability, and cell death [10,11]. The following section will address several key *in vivo* studies in which siRNA were applied as a potential therapeutic tool against brain hemorrhages (Table 1).

**Table 1:** Published *in vivo* usage of RNA interference as a therapeutic tool in Nontraumatic Brain Hemorrhage Models.

Reference	Targeted Protein	Pathophysiological Pathway Targeted	Injury Model	Delivery Method	Delivery Timepoint	Result
[12]	CHOP	apoptosis	Endovascular perforation of MCA	ICV	24 h prior	↓ edema ↓ BBB disruption ↓ behavior deficit
[13]	CHOP	apoptosis	Endovascular perforation of MCA	ICV	24 h prior	↓ cell death Reversed detrimental phenotypic changes of the blood vessels
[14]	PUMA	apoptosis	Endovascular perforation	ICV	Immediately after	↓ cell death ↓ edema ↓ behavior deficit ↓ BBB disruption ↓ mortality rate
[15]	VAP-1	neuroinflammation	Collagenase Injection	ICV	48 h prior	↓ edema ↑ behavior ↓ microglial activation ↓ proinflammatory molecule secretion

#### 2.1.2. *In Vivo* siRNA Studies

In a rat model of SAH, siRNA against *C/EBP homologous proteins (CHOP*) were administered 24 h prior to injury [12], to prevent CHOP-mediated cell death of endothelial cells. Rats pre-treated with siRNA against *CHOP*, before endovascular perforation of the middle cerebral artery, showed decreased mortality, improved neurological recovery, decreased BBB disruption, and decreased edema. Improved outcomes were probably due to decreased Bim (Bcl-2-interacting mediator of cell death), increased beclin-2, and decreased cleaved caspase-3, leading to decreased cell death and better neuronal cell survival [12]. Additional studies in this model characterized the effect of pretreatment with siRNA against *CHOP* on phenotypic changes in cerebrovascular blood vessels, showing decreased diameter and wall thickness in the basilar artery in the treated rats after SAH, along with decreased apoptosis as previously observed [13]. Because narrowing of the artery and thickening of the wall can lead to further damage and cause cerebral vasospasm, decreases in this phenotype induced by siRNA against *CHOP* indicates that pre-treatment is beneficial.

In another model of rodent SAH, siRNA against *p53 up-regulated modulated regulator of apoptosis (PUMA)* also showed decreased mortality, improved neurological scores, decreased BBB disruption, and decreased cerebral edema [14]. In the normal cascade of SAH, *PUMA* may induce microvascular changes and damage resulting in cellular apoptosis. By contrast, siRNA against *PUMA* provided an effective treatment to reduce BBB disruption and edema, thereby translating into improved outcomes [14].

In a study indirectly targeting the cell death pathway, siRNA against *vascular adhesion protein 1 (VAP-1)* was utilized in a collagenase injection injury model in mice, which mimics ICH [15]. Under neuroinflammatory conditions, VAP-1 has a role in systemic immune cell migration and infiltration across the BBB into the brain parenchyma. As predicted, inhibition of VAP-1 with siRNA attenuated cerebral edema and improved neurological recovery 24 h post-injury [15].

Notably, siRNA targeted proteins presented here in non-traumatic hemorrhage injury models have a profound effect on injury cascades involving blood vessels in one way or another. This is somewhat expected, since non-traumatic hemorrhages are by definition caused by aneurism or rupture of cerebral blood vessels. Thus, to contain further damage, limiting endothelial cell death and detrimental phenotypic transformation [16] is a logical strategy against SAH and ICH. A limitation of these *in vivo* animal studies was the delivery route of the siRNA via intracerebroventricular (ICV) injection. ICV allows for direct access of siRNA to the injury site, but may be an impractical and invasive approach for clinical practice, as described later during discussion of siRNA injection routes.

### 2.2. Cerebral Ischemic Stroke

#### 2.2.1. Background

According to the American Heart Association, stroke is the number one cause of chronic disability and the fourth leading cause of death in the U.S. There are nearly 800,000 cases occurring annually [17], causing a financial burden of about 63 billion U.S. dollars [18]. The majority of these cases are ischemic in nature, and the rest are hemorrhagic [17].

Cerebral ischemic stroke is caused by an occlusion of a cerebral blood vessel, which triggers decreases in blood flow, oxygen, and nutrients to desired brain regions. The lack of cerebral blood flow impairs normal cellular function and promotes neuronal death. The vascular system also becomes fragile in the absence of oxygen/glucose during the period of occlusion, and receives a second wave of detrimental shearing stress during reperfusion. Thus, prompt treatment following the onset of stroke is highly desirable, as each passing moment increases the likelihood of irreversible damage to the nervous tissue [19]. However, recombinant tissue plasminogen activator (rtPA) is currently the only thrombolytic molecule administered during acute cerebral infarction. It provides clinical benefit in terms of survival and neurological outcome, despite a narrow time window and strict patient eligibility, as rtPA administration may induce potentially fatal bleeding in some patients [20,21,22,23]. Evidently, other therapeutic drug treatment options are urgently needed.

The pathophysiological and molecular cascade of events occurring after ischemic stroke has been researched extensively, and several proteins of interest are becoming available as therapeutic targets for improvements of post-ischemic recovery. An agent such as siRNA that has the ability to specifically knockdown proteins of interests is greatly desired in this field as well. In the following section, we will review several promising *in vivo* studies published in the last few years, which all employ various siRNA as therapeutic tools against ischemic stroke (Table 2).

#### 2.2.2. *In Vivo* Studies

Several studies report siRNA administration in models of transient middle cerebral artery occlusion (tMCAo) targeting different molecular pathways involved in post-ischemic pathophysiology, from apoptotic cell death to inflammation [24,25]. Early on, researchers focused on neuroprotective strategies to limit the spread of apoptotic cell death pathways which normally develop following an ischemic event. The first study used siRNA against Beclin1, a protein responsible for cell autophagy and apoptosis [26]. Rats treated with Beclin1 siRNA showed decreased infarct volume and improved neurological outcome, resulting from decreased apoptosis and increased neurogenesis [26]. Similarly, in a rat stroke model of endothelin 1 injection, siRNA against *caspase-3* showed decreased apoptosis and improved forelimb functional recovery [27]. Similarly, a study published in the same year targeted another protein involved in the apoptotic cascade, the apoptosis signal-regulating kinase 1 (Ask1) [28]. As the name implies, Ask1 plays a role in apoptosis and cell differentiation, and mice treated with Ask1 siRNA showed decreased infarct volume and decreased cell death [28]. However, it is important to note that in all of these studies, neuroprotective siRNA delivery occurred before the ischemic event, which limits translational approaches in a clinical setting.

In an effort to directly target vascular compartments, siRNA against the *protease-activated receptor-1 (PAR-1)* was used in mouse models of tMCAo. PAR-1 is involved in the blood coagulation pathway, and serves as another viable option to the thrombolytic strategy alongside rtPA. siRNA against PAR-1 seven days prior to injury resulted in a decreased infarct volume at 24 and 72 h post injury, and a significantly lower neurological deficit at the same timepoints. PAR-1 down-regulation also resulted in decreased levels of heat-shock protein-70 (HSP70) and microtubule-associated protein-2 (MAP2) [29]. Interestingly, PAR-1 is also present in other cell types such as astrocytes and neurons, and previous studies suggested that high thrombin levels could be detrimental if induced via PAR1 [30,31]. Therefore, orchestrating the optimal levels of several cellular targets within the neurovascular unit (NVU) may have overall benefits, provided we can resolve the caveat of pre-injury siRNA delivery.

In addition to hemorrhage, hypoxia is a key ischemic symptom resulting from decreased oxygen delivery, which has tremendous effects on the downstream injury cascade. One key protein is hypoxia-inducible factor-1α (HIF1α), which is induced after stroke and is involved in NVU dysfunction. By targeting HIF1α with a specific siRNA at 1h after tMCAo in rats, treated animals exhibit decreased BBB disruption, decreased mortality, and decreased infarct volume, which was associated with behavior improvements. These results were concomitant with lower protein expression of p53, cleaved caspase-3, and vascular endothelial growth factor [32].

Another important injury mechanism is neuroinflammation. In a recent study, repeated siRNA administration against one neuroinflammatory pathway provided the opportunity to study both acute and lasting effects of siRNA administration [33]. The authors targeted G-protein coupled receptor 17 (GPR17), a protein with proposed roles in post-ischemic neuroinflammation. Previous studies in which GPR17 was inhibited via antisense oligonucleotide showed beneficial effects after ischemic stroke [34,35]. Zhao *et al*. specifically examined the effect of siRNA against GPR17 on microglial activation at both the acute and chronic post-injury stages, finding decreased microglial activation at 14 d but not at 24 h post injury [33]. These findings are interesting in the context of a hypothesized dual role of post-injury microglial activation, in which acute activation after injury is beneficial but could be detrimental if lasting for extended long-term periods [36,37]. Another important player during the post-ischemic neuroinflammatory phase is the high mobility group box 1 (HMGB1), which is secreted by necrotic cells to orchestrate functions such as inflammatory cell recruitment and migration [38]. Intracortical injection of siRNA against HMGB1 was neuroprotective after ischemic stroke through attenuation of microglial activation and neuronal apoptosis [39,40]. In subsequent experiments, intranasal administration of siRNA against HMGB1 in a rat model of tMCAo resulted in a significant knockdown of HMGB1 in various regions of the brain, but not in the liver, lung, kidney, or heart [41]. Furthermore, this effective knockdown resulted in improvements in behavior testing [41]. An important point to address for this particular study is its drug delivery method. Although the siRNA was still administered pre-injury, the group used an intranasal delivery of siRNA, thus side-stepping from one of the greatest obstacle for drug delivery to the brain: The BBB. This will be covered again later, but suffice it to say that intranasal delivery of drugs is one of the most promising tools, not only for siRNA delivery to the brain, but for other drugs as well [42,43,44,45].

In the stroke studies presented above, it is interesting that positive benefits were observed for so many different and unique siRNA targets. Evidently, several post-ischemic pathophysiological pathways play a vital role in preservation of tissue phenotype, cellular remodeling, functional integrity of the neurovascular unit, and beneficial outcomes on several behavioral endpoints. Aside from the fact that siRNA injections occurred prior to the injury, these results are encouraging for development of future combinatorial siRNA therapies, in which administration of siRNA targeting two or more of these proteins involved in different pathways may provide additive benefits to post-injury recovery paradigms.

**Table 2:** Published *in vivo* usage of RNA interference as a therapeutic tool in Ischemic Stroke Models.

Reference	Targeted Protein	Pathophysiological Pathway Targeted	Injury Model	Delivery Method	Delivery Timepoint	Result
[27]	Caspase-3	Apoptosis	ET-1 Injection Rats	Intracerebral cortex Injection	24 h Prior	↓ TUNEL ↑ behavior outcome (significant only at 24 h PreInjury)
					24 h Post	
[26]	Beclin1	Apoptosis	Transient MCAO Rats	Lateral Ventricle Injection	7 d Prior	↓ infarct volume ↑ neurological outcome
[28]	Ask1	Apoptosis	Transient MCAO Mice	Osmotic Minipump Ventricle	Continuously 3 d Prior	↓ infarct volume ↓ TUNEL
[29]	PAR1	Coagulation Cascade	Transient MCAO Rats	Lateral Ventricle Injection	7 d Prior	↓ infarct volume ↑ neurological outcome
[32]	HIF1α	Hypoxia Induced Cascade	Transient MCAO Rats	Intraparenchyal Injection	<1 h Post	↓ mortality ↑ neurological outcome ↓ Infarct Volume ↓ BBB disruption ↓ cell death
[33]	GPR17	Microgliosis (neuroinflammation)	Transient MCAO Rats	ICV Injection	Once daily from 2 d prior to 7 d post and then every other day from day 8–14	24 h post injury ↓ neurological dysfunction ↓ infarction ↓ neuron loss
						14 d Post injury ↓ brain atrophy ↓ neuron loss ↓ microglial activation
[41]	HMGB1	Neuroinflammation	Transient MCAO	Intranasal	1 h Prior	↓ infarct volume ↑ behavior

### 2.3. Traumatic Brain Injury

#### 2.3.1. Background

Traumatic brain injury (TBI) has been termed a “silent epidemic” in the U.S. and directly affects at least 1.7 million people, causing a huge financial burden for patients, families, and communities at large [46]. TBI is characterized by primary and secondary stages of injury. Primary injuries are the result of the direct and immediate mechanical disruption of the brain tissue, and secondary injuries are the result of an indirect and more delayed downstream mechanisms arising from the primary injuries [47]. The severity of primary injuries can and has decreased in recent years due to increased public and legislative awareness concerning preventive measures. For example, there is increased use of protective helmets when engaging in activities at high-risk for TBI such as riding bicycles, playing sports, or military combat. Since only preventive or preemptive tactics can be applied to lessen the damage from primary injury, research in the development of efficient post-injury therapeutic treatments should focus on targeting secondary injury cascades [48]. Secondary injuries involve a myriad of pathophysiological events such as edema, BBB disruption, neuroinflammation, and cell death. Despite the harm that TBI causes at the individual, communal, and national level, no effective pharmacological treatment exists to date. However, as noted for other types of acute brain injuries, siRNA contains the desired characteristics of specificity and potency which could be a beacon of hope for the many who suffer from TBI. In this regard, there are a few notable *in vivo* studies in which siRNA were employed as a therapeutic option for TBI (Table 3).

#### 2.3.2. *In Vivo* Studies

Cerebral edema is one of the major landmarks and a defining feature of juvenile and adult TBI, and to date there are no perfect treatments to effectively prevent edema formation or its consequences on secondary injury cascades. Thus, it is noteworthy that the few available and recent studies chose to develop siRNA targets in molecular pathways of post-injury edema formation. First, in a model of cold-cortical injury, often used to mimic the process of vasogenic edema after TBI, injection of siRNA against *interleukin-6 (IL6)* one hour post injury caused a significant decrease in lesion volume at seven days post injury [49]. The authors hypothesize that lesion volume reduction is due to increased neoangiogenesis resulting from increased HIF-2α, which is normally inhibited by IL6. However, IL6 may directly impact edema formation, thus we cannot exclude the direct effects of IL6 on neuroinflammatory pathways after TBI [50]. In a similar model, Campbell *et al*. showed that siRNA against claudin-5, a tight junction protein providing BBB stability, was able to decrease edema, reduce lesion size, and lead to significantly lower scores on a scale of neurological severity and impairment scoring [51]. By targeting a tight junction protein known for providing structural integrity to the BBB, there is a risk to weaken and disrupt the barrier, causing more long-term damage. However, Campbell *et al*. showed improvements in post-injury recovery, highlighting that a weakened BBB can aid in water extravasation out of the brain parenchyma, thus supporting edema resolution.

An important player in post-injury edema is the aquaporin water channels. Aquaporin 4 (AQP4) is a water channel protein expressed abundantly in the brain, especially in the perivascular astrocyte endfeet hypothesized to play a central role in the cerebral edema process in stroke [52] as well as in TBI [53]. In our group, we recently developed siRNA targeting AQP4 (siAQP4). In a model of controlled cortical injury, siRNA against *aquaporin 4* led to beneficial results after injury as well [54]. By studying the acute and chronic effect of siRNA administration post-TBI in rodents, we observed several notable features highlighting siAQP4 as a promising clinical treatment. Even though AQP4 levels were consistent between siAQP4 treated and control saline groups at two months after injury, beneficial effects could still be observed in the form of improved recovery and improved neuronal survival for treated animals. This is very important when considering AQP4, as a biphasic role of AQP4 has been hypothesized for this protein, where acutely AQP4 is involved in edema formation—water entry into the brain parenchyma, but chronically involved in edema resolution—water exiting from the brain parenchyma. Therefore, an acute down-regulation of AQP4 is desired, but chronic down-regulation may prove detrimental. In our model, post-injury siAQP4 administration effectively achieved this dual need; we observed that siAQP4 administered acutely after injury was beneficial, but RNA interference against AQP4 did not last long enough to provide chronic impairment when edema resolution became a priority.

Another key pathophysiological phenomenon that occurs after brain injury is cell death resulting from over-accumulation of intracellular calcium [55]. Although this calcium related cell death has been well known for decades [56], molecular mechanisms behind this phenomenon are still in the discovery phase. Niu *et al*. investigated whether TBI induced changes in intracellular calcium release mediated by the protein Frizzled-2, and whether siRNA against *frizzled-2* would result in decreased intracellular calcium accumulation [55]. Using a weight-drop model of traumatic brain injury, rats injected with siRNA against *frizzled-2* exhibited decreased intracellular calcium in the injured hippocampus [55], thus providing evidence for the possibility of attenuating the pathophysiological consequences after TBI by suppressing calcium overload. Although this study did not report behavioral benefits after the treatment, one can hypothesize that a decrease in the pathological over-accumulation of calcium would most likely result in improved recovery after injury in hippocampal dependent tasks.

**Table 3:** Published *in vivo* usage of RNA interference as a therapeutic tool in traumatic brain injury models.

Reference	Targeted Protein	Pathophysiological Pathway Targeted	Injury Model	Delivery Method	Delivery Timepoint	Result
[51]	Claudin-5	Edema	Cold Induced Mice	Tail Vein Injection	<1 h Post	↓ lesion size ↑ cognitive outcome
[49]	Int6	Angiogenesis	Cold Induced Rats	Internal carotid Artery	1 h Post	↓ lesion
[55]	Fzd2	Ca^2+^ Accumulation	Weight Drop Rats	Direct Hippocampal Injection	48 h Prior	↓ Fzd2, Wnt5a, p-CaMKII ↓ intracellular Ca^2+^
[54]	AQP4	Edema Formation	Controlled Cortical Injury	Intracortical Injection	Immediately after injury and 2 d after injury (2 injections)	Acutely, ↑ motor function ↑ neuronal survival ↓ BBB disruption ↓ edema Chronically, ↑ memory ↑ neuronal survival

Remarkably, all four TBI studies described above have siRNA targets against non-neuronal cells. On one hand, the paucity of these studies leaves several options open for further exploration. On the other hand, this strategy supports the notion of targeting support cells in the neurovascular unit as they work in a collaborative fashion to maximize neuronal benefit and behavioral improvement. siRNA targeting non-neuronal cells for therapeutic purposes is certainly an intriguing approach, especially considering the complexity of interactions between different cell types in the brain and their influence on one another.

### 2.4. Overall Summary of the *In Vivo* Acute Brain Injury Studies

Out of the 15 studies using siRNA as a potential drug, six studies applied siRNA targeting proteins involved with the apoptotic cell death pathway, three studies directly targeted neuroinflammation, and the remaining studies covered other pathways such as edema, calcium overload, coagulation, *etc.* That being said, since many of these injury cascades affect each other, by directly targeting a specific protein of an injury cascade, beneficial effects in other areas could be observed as well. For example, reducing edema may reduce BBB disruption and eventually attenuate cell death. Alternatively, some proteins may be involved in multiple injury pathways such as the GPR17, which seem to affect both neuronal survival and microgliosis [33,54]. Therefore, although primary pathways were catalogued for each targeted protein, the broader effect is most often diverse and part of a cascade. On the same note, it is very exciting to observe that acute administration of siRNA could have long-term beneficial effects (Fukuda *et al*. JCBFM, accepted for publication) and holds much clinical promise. Furthermore, although acute brain injuries differ greatly in their etiology, all of them rely on the integrity and function of the same cerebral vasculature and share common injury pathways such as cell death, neuroinflammation, edema, and BBB disruption. Thus, effective siRNA treatments for one injury model may crossover quite easily for other injury models as well.

One major limitation of all of these studies is the applicability from the bench to the patient’s bedside. In fact, nine studies out of the 15 administered siRNA prior to the injury, five studies administered siRNA after the injury, and one study carried out two different experiments in which the animals were injected either 24 h prior to injury or 24 h post-injury [27]. However, this group did not observe a statistically significant improvement between groups when siRNA was administered after injury, whereas a statistically significant improvement was observed when the siRNA was administered 24 h prior to injury [27]. All five studies using post-injury siRNA administration did so within the first hour after injury and observed several beneficial outcomes. Overall, regardless of siRNA delivery time with respect to the injury, all studies showed improvements demonstrating the efficacy and potential of siRNA as a therapeutic tool. Unfortunately, pre-treatment with siRNA still presents a problem and does not mimic clinical settings and even when administered after injury, it is logistically difficult to directly translate this to patients within one hour after onset of an acute brain injury. As demonstrated by Al-Jamal *et al*., pre-treatment and post-treatment could yield different effects, and siRNA administered immediately after injury and a few days after injury may also have differing efficacy [27]. Therefore, once theoretical confirmation of the efficacy of specific siRNAs are achieved *in vivo* through pretreatment or acute post treatment. The logical next step would be to test for efficacy even when the siRNA has been administered at least a few hours after injury, thus mimicking timelines expected in clinical practice.

Finally, and perhaps most importantly for clinical translation, we categorized the delivery method. Delivery of siRNA is perhaps the largest obstacle that stands in the way of clinical usage against acute brain injuries because under normal conditions the siRNA cannot cross the BBB. Out of the 15 studies, eight studies employed intracerebroventricular injection, four studies employed intra-parenchymal injection, and one study administered via the internal carotid artery. One research group was able to down-regulate expression of claudin 5 with a tail injection [51]. This was achieved not because of the delivery method, but due to the localization of the targeted protein as part of the structural network forming the BBB. Thus, Campbell *et al*. did not need to cross the BBB, but rather, targeted the BBB directly. Furthermore, the authors did not observe major histological abnormalities in the major organs, or any significant differences in hematological analysis. However, because claudin-5 is present in other organs, it is worthwhile to note that siRNA transfection was effective in the liver and lung endothelial cells as well [51]. Another group employed the intranasal delivery method for HMGB1 with success, which allowed the siRNA to completely bypass the BBB and directly access the brain [41]. This method is most likely suitable for all siRNA therapies, and serves as a notable player in the future of siRNA in both basic science and clinical applications. A brief description of the advantages and disadvantages of each of the siRNA delivery method has been made in Table 4 (Table 4).

**Table 4:** Advantages and disadvantages of each delivery method of siRNA.

Method of delivery	Advantages	Disadvantages
Intra-cerebroventricular	Directly in the brain; Distribute in all brain	Difficult to apply in clinic; Dilution of the siRNA and potential degradation; Off target effects on other brain regions
Intra-vascular, tail vein and carotid	Possible in clinic	Difficulties to cross BBB; Off target effects on other organs (*i.e.*, Liver); Dilution of the siRNA; Increase chance of siRNA degradation
In the brain structures: Cortex and hippocampus	In the targeted brain region; Small amount of siRNA	Difficult to apply in clinic; Mechanical lesion by the injection; Neuroinflammation
Intra-nasal	Target the brain; Diffusion along the perivascular space; Possible in clinic; Accommodate large molecules	Potential dilution of siRNA and degradation; Off target effects on lungs and other brain regions

## 3. Future Direction

The ongoing trend observed throughout the studies highlighted in the current review show a promising future for the multiple siRNA targets. Several cascades may be able to improve brain injury recovery through various different pathophysiological pathways such as apoptosis, edema formation, neurogenesis, angiogenesis, and calcium accumulation. siRNA seems to be readily available for a wide range of cell types and pathophysiological pathways, thus providing a seemingly endless array of possibilities.

As previously mentioned, a clear obstacle for drug delivery to the injured brain is the BBB, which protects the brain from unwanted systemic circulatory toxins and pathogens by blocking the entry of molecules that are lipid-insoluble, macromolecules, and naked molecules that are not carrier-mediated or receptor-mediated, owing to the tight junctions [57]. While this is an essential mechanism that ensures the protection of the CNS, under pathological conditions these same features stand as unique difficulties to be overcome by clinicians and researchers. Proper diffusion is needed to the targeted cells (astrocytes/neurons/endothelium), efficiency is required in cell transfection, and potential degradation of the siRNA should be avoided.

Other obstacles or challenges to be considered are more generally pertinent to siRNA therapy targeting any types of organs and tissues due to the inherent characteristics of the siRNA. Such characteristics include the varied half-life of siRNA, siRNAs as anions, possible off-target gene silencing, immune activation, and the degradation of siRNA by RNAses. Several of these issues will be discussed in detail in the remainder of this review paper, along with promising techniques that are being utilized in the lab to overcome these difficulties. However, each of the above mentioned features will be briefly discussed. First, the half-life of any given drug is an important factor that determines the available timeframe that the therapeutic molecule has in reaching the targeted region or cell to execute its therapeutical role. For siRNA, naked siRNAs without any modifications, like coating, has a short half-life [58,59], so prolonging the half-life in order to achieve maximum effect is an essential part in developing siRNA therapeutics. The anionic property of naked siRNA, combined with its large molecular size makes crossing bilipidic membranes difficult, especially across the BBB. Indeed, intravenous injection of radiolabeled naked siRNA in rats showed the brain to be the organ that had the least amount of siRNA [60]. Thus, efficient systems of delivery must be incorporated in order to overcome this transport issue. Although siRNA is specific in suppressing the targeted mRNA, there are still possibilities for off-target gene silencing [61]. Sometimes, through the interferon pathway, siRNAs may activate unwanted immune responses that may cause the detrimental effect to overshadow the therapeutic benefits. Regarding off-target gene silencing and adverse immune responses, a careful design of the siRNA strand is required. Although, one cannot discard the fact that with an improved specificity of delivery to cells of interest, adverse immune responses in other areas of the body will be lessened. Finally, siRNA could be enzymatically degraded by endogenous RNAses, even after reaching the targeted cell. This is due to the endosomes (different to exosomes which will be discussed later on in the review) and lysosomes, which are major players in the endocytotic pathway which allows siRNAs to be internalized in the cells. Unfortunately, the ultimate fate for these molecules inside endosomes is hydrolytic degradation, caused by various RNAses. Thus, the siRNA needs to “escape” from these endosomes to avoid degradation prior to achieving its effect by attaching to the target mRNA [58].

Therefore, proper diffusion to the targeted cells (astrocytes/neurons/endothelium) is needed, efficiency is required in cell transfection, and potential degradation of the siRNA should be avoided. So far, most of the studies involved injection of the siRNA directly into the brain, which is invasive and most often involves a craniotomy. Also, the siRNA is usually associated with a transfection reagent to help the siRNA enter the cells. In certain situations when craniotomy is needed as a surgical method to decrease brain swelling after brain injury, this approach could be proposed in parallel to the surgery procedure. However, not all injuries would warrant a craniotomy, and siRNA efficiency may require multiple injections, which could have additional complications with this approach. Thus, non-invasive modes of drug delivery are required in order for the siRNA to become a therapeutic option for acute brain injuries. One potentially promising tool to achieve selective and efficient RNA interference targeting inside the brain is through the intranasal route of drug delivery, which is efficient to deliver peptides as well as cells [62].

### 3.1. Intranasal Drug Delivery

Intranasal drug administration is an attractively simple method of choice which is non-invasive, time-efficient, cost-effective, and provides procedural ease. Dr. Frey and his collaborators are one of the pioneers in the field of intranasal drug delivery to brain diseases such as stroke and Alzheimer’s disease, showing numerous promising results in which a wide variety of compounds such as insulin like growth factor and deferoxamine provided efficient improvements in recovery with no significant side-effects [45,63,64,65,66,67,68]. Because the intranasal cavity has access to the brain and is unhindered by the BBB, even siRNA, which will not cross the BBB under normal circumstances due to its high charge and molecular weight, can reach the brain cells through the intranasal route. There is already at least one study in which siRNA was administered intranasally in a rat stroke model, and beneficial results were obtained [41]. Other studies have observed siRNA administered via the intranasal pathway to effectively travel to the olfactory nerves and olfactory bulb [69], and raphe nuclei targeting the serotonergic neurons [70]. Certain proteins which may be expressed abundantly and/or systemically could provide additional challenges when considering the potential effect that down-regulation of proteins in undesired organs could provide, especially in the respiratory organs. This potential side-effect should be considered and investigated because some *in vivo* experiments have used intranasal delivery of RNA interference as treatments to target the lungs [71,72]. Finally, even when the siRNA has successfully breached the BBB and reached the parenchyma, it must be diffused successfully and travel far enough to the desired regions of the brain and target the desired cells. Because brain tissue is not homogenous and there is a varying level of tortuosity due to the structural gradients formed by the cells and extracellular space, pharmacokinetic studies may provide assurance of effective delivery of siRNA within the brain [73]. However, the work performed by Kim *et al*. shows promise because intranasally administered siRNA in a tMCAo model of rats showed marked reduction of the target protein only in the brain, but not in peripheral organs including the lungs [41]. Within the first hour after intranasal administration, siRNA was observed in the cytoplasm of neurons, astrocytes, and microglial cells in the frontal cortex, striatum, amygdala, and hypothalamus and was maintained for at least 12 h after injection [41].

### 3.2. Exosomes, Microvesicles and Nanoparticles

Another potential means of transporting the siRNA to the brain across the BBB are exosomes [74,75]. Exosomes are endogenously produced vesicles that have a diameter of about 40–120 nm, with a lipid bilayer membrane that allows separation of the internal content of the exosome from the external environment. One of the physiological roles of exosomes is the transfer of RNA and miRNA [75]. The advantage of using exosomes as the transporting agent for siRNA is their minimal toxicity due to their endogenous origin. A recent study performed by Alvarez-Erviti *et al*. shows a very promising result in which a tail vein injection of siRNA against GAPDH loaded in central nervous system-specific rabies viral glycoprotein (RVG)-targeted exosomes led to a specific inhibition of the target protein only in the brain with no effect on other organs. Furthermore, injection of the RVG-targeted exosome did not result in overt secretion of proinflammatory molecules, no side-effects were reported, and the efficacy of the delivery or the siRNA itself did not decrease over multiple deliveries [74]. RVG conjugated exosomes can be a useful tool on their own, but when combined with the intranasal delivery, could form a formidable combination in which safe, specific targeting of brain tissue will be achieved as well as a mechanism to target one cell more than another (*i.e*., astrocyte *vs.* neurons). Moreover, the exosome also brings protection to the siRNA from potential degradation [76], which will improve the efficiency of the drugs by prolonging the half-life in addition to correct targeting. Along the same lines, other potential delivery vehicles include extracellular membrane vesicles (EMV), known as a new cell-to-cell genetic communication machinery [77,78]. The synthetic EMV or liposomes are well studied and suitable as a drug nanodelivery system. This natural or synthetic lipid bilayer forms spherical microvesicles, which could easily cargo and deliver the siRNA to specific cells as proposed for the exosomes. A limitation may be the high immunogenicity of liposomes, which is now likely to be reduced with new understanding of the biology of the EMV and the new generation of microvesicles (for details see review by Lai and Breakefield [78]). However, the immunogenicity is likely to be higher using the viral transfection classically proposed for any gene therapy (including siRNA transfection), limiting the repetition of the injections, frequently needed in the siRNA approach [79].

A third category of delivery methods that are being researched is the utilization of nanoparticles, synthetic organic polymers and inorganic materials, which provides potential solutions to counter degradation of siRNA, transport across various barriers within the body including the BBB, and better uptake to specific tissues or cells within the target organ with coupling with one or multiple target ligands. The relatively novel field of nanotechnology has aided the field of pharmacology, especially in the area of efficient delivery of therapeutic agents, as evidenced in the field of oncology [80,81]. Anti-cancer therapeutics have benefited from reduced immunogenicity and toxicity and increased half-life due to nanotechnology [58,80,81]. Because these are also issues that challenge the clinical translation of siRNA technology, successful incorporation of nanoparticles will aid in the widespread usage of siRNA as a therapeutic option. Zhou *et al.* has recently published a very informative review covering the various nanoparticle based strategies being employed to improve siRNA delivery under pathological conditions as well as some of siRNA coupled nanoparticles under clinical trials [58]. Some of the strategies discussed are bacteriophage phi29 Packaging RNA based nanoparticles, nucleic acid aptamer-based nanoparticles, protein or peptide based nanoparticles, mesoporous silica nanoparticles, polyethyleneimine, cyclodextrin polymers, cationic dendrimers, and liposome-based nanoparticles [58]. These new developments are providing hope for the correct delivery of siRNA for treatment in brain injuries.

Finally, although not a delivery method for siRNA, microRNAs (miRNA) comprise another family of important non-coding RNA molecules that have a similar function to siRNAs. Briefly, miRNA can affect post-transcriptional gene expression by either inhibiting translation or causing degradation of the mRNAs that code for specific proteins [5]. Numerous miRNAs have been identified so far in mammals, and thus far, several research groups have identified tissue-specific miRNA in the brain compared to other organs of the mammalian body [82,83,84]. miRNA expressions have been documented to change after models of TBI and ischemia [83,85,86,87], where different sets of miRNA showed changes at different timepoints after the injury. With these observations, miRNA also seem to be an interesting target or tool for eventual treatment strategies in patients suffering from acute brain injuries. However, there is a key difference between siRNA and miRNA as a potential therapeutic tool. The majority of miRNA in animals have been shown to affect post-transcriptional gene regulation through translational depression, which is caused by an imperfect match between the miRNA and mRNA. This imperfect complementarity allows a single type of miRNA to have an effect over potentially hundreds of mRNA and proteins. While this is an essential mechanism in the endogenous gene regulatory network and pathways, at first glance it may not be a desirable characteristic when making drugs that must target specific proteins to avoid unwanted side-effects. However, although one miRNA could potentially target numerous proteins, if the targeted proteins all work in concert for a specific function such as cell death or neuroinflammation, targeting a single miRNA may result in an effect equivalent to turning off a master key switch to effectively and greatly attenuate the injury cascade by targeting multiple proteins at the same time. Thus, although more research needs to be done, miRNA may also provide a unique opportunity other than siRNA therapy.

## 4. Conclusions

Using siRNA to study and treat acute brain injuries such as stroke and TBI is a very promising modality of therapy that could lead to a global break-through in improving patient recovery. In order to achieve this, more *in vivo* studies must be conducted in which post-injury administration of siRNA targeting various proteins of the brain is linked with behavioral improvements. Furthermore, to get the most out of the potency of the siRNA, brain delivery methods must be refined on several fronts. Despite the obstacles, siRNA awaits like the sword-in-the-stone for the right circumstances of person, place, and time, and novel mechanisms are already underway. Delivery via intranasal administration and using exosomes/microvesicles represent some of the most innovative and promising methods to safely bypass the BBB and reach specific protein targets in the brain.

## Abbreviations

small interfering RNA: (siRNA)
blood-brain barrier: (BBB)
traumatic brain injury: (TBI)
subarachnoid hemorrhage: (SAH)
intracerebral hemorrhage: (ICH)
middle cerebral artery: (MCA)
middle cerebral artery occlusion: (MCAo)
intracereboventricular: (ICV)
neurovascular unit: (NVU)
